# The recruitment mechanisms and potential therapeutic targets of podocytes from parietal epithelial cells

**DOI:** 10.1186/s12967-021-03101-z

**Published:** 2021-10-21

**Authors:** Lihua Ni, Cheng Yuan, Xiaoyan Wu

**Affiliations:** 1 Department of Nephrology, Zhongnan Hospital of Wuhan University, Wuhan, 430071 People’s Republic of China; 2 Department of Gynecological Oncology, Zhongnan Hospital of Wuhan University, Wuhan, 430071 People’s Republic of China

**Keywords:** Parietal epithelial cells, Podocyte, Progenitors, Transdifferentiate, Recruitment

## Abstract

Podocytes are differentiated postmitotic cells which cannot be replaced after podocyte injury. The mechanism of podocyte repopulation after injury has aroused wide concern. Parietal epithelial cells (PECs) are heterogeneous and only a specific subpopulation of PECs has the capacity to replace podocytes. Major progress has been achieved in recent years regarding the role and function of a subset of PECs which could transdifferentiate toward podocytes. Additionally, several factors, such as Notch, Wnt/ß-catenin, Wilms’ tumor-1, miR-193a and growth arrest-specific protein 1, have been shown to be involved in these processes. Finally, PECs serve as a potential therapeutic target in the conditions of podocyte loss. In this review, we discuss the latest observations and concepts about the recruitment of podocytes from PECs in glomerular diseases as well as newly identified mechanisms and the most recent treatments for this process.

Podocytes are terminally differentiated cells and generally do not replicate, presenting a major obstacle to their restoration [1–4]. Replacing lost podocytes is a therapeutic opportunity to limit and reverse glomerular scarring and proteinuria. Previous studies have demonstrated that some glomerular parietal epithelial cells (PECs) act as progenitors of podocytes in healthy glomeruli and follow a decreased number of podocytes under healthy and disease conditions [5]. Romagnani’s group was the first to characterize progenitor cells of the Bowman’s capsule, providing novel insight into glomerular physiology [6–8]. In Bowman’s capsule, they isolated and characterized CD24^+^CD133^+^ PECs as multipotent progenitor cells. These cells could be triggered to generate mature and functional tubular cells in human. Several signaling pathways regulate PEC proliferation and differentiation toward podocytes [9]. In this review, we summarize the current progress about the roles and functions, involved mechanisms and potential therapeutic targets for podocyte recruitment from PECs.

## Introduction of podocyte injury

Podocytes maintain the glomerular filtration barrier. Podocyte injury causes proteinuria and terminally glomerulosclerosis. The critical factor preventing podocyte injury may be the lack of regenerative capacity in podocytes.

**Fig. 1:**
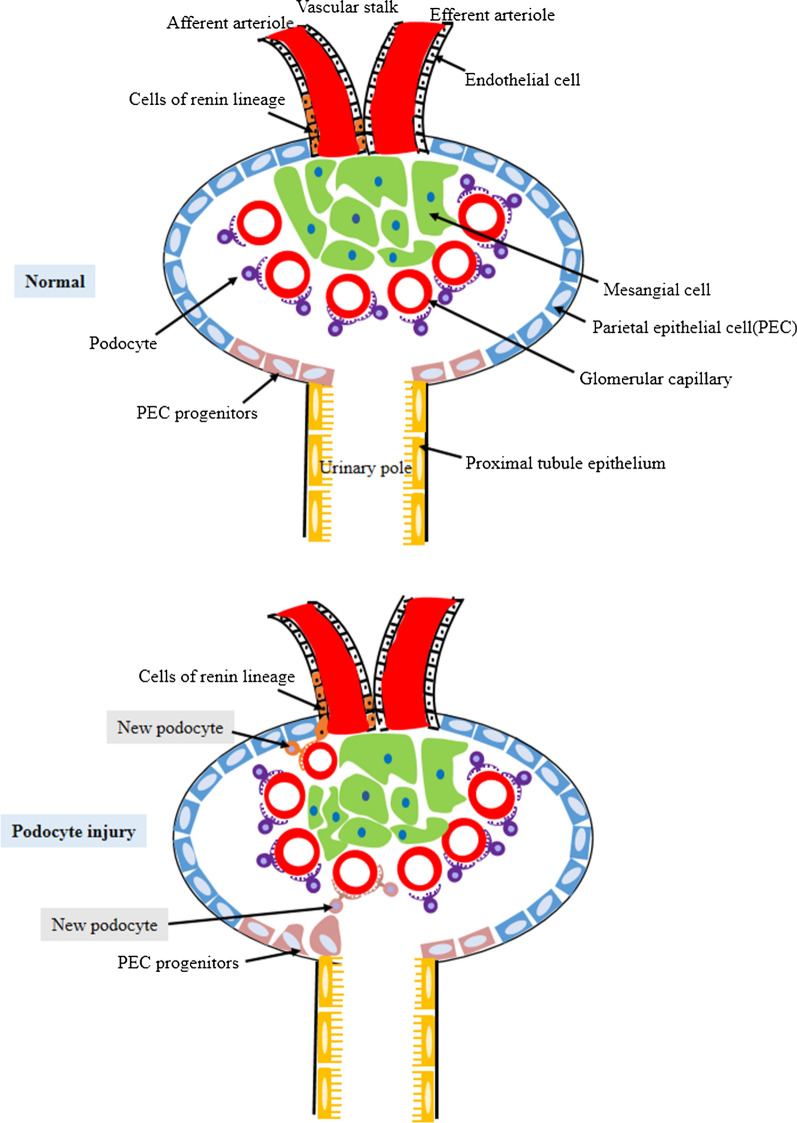
Schema representing the fate of PECs and cells of renin lineage as podocyte progenitors. Both of them could acquire podocyte-like qualities. PECs: parietal epithelial cells

Some podocyte progenitors were activated in the human Bowman capsule after podocyte injury (Fig. 1). PECs are the well known source for podocyte progenitors. Besides, the cell of renin lineage (CoRL) is a new-found candidate for the podocyte progenitor.

A current study [10] suggests the CoRL transdifferentiate to podocytes in acute FSGS, 5/6 nephrectomy-induced chronic kidney disease, and uninephrectomy models. However, only podocyte loss, rather than a decrease in nephron mass, triggers CoRL of juxtaglomerular origin to move to the injury site.

Interestingly, the podocyte depletion paradigm have raised concerns these years. Puelles et al. [11] collected kidneys at autopsy from Caucasian American adults, and they suggested that older age and hypertension are associated with podocyte depletion, which is more obviously when both older age and hypertension are present.

Above all, podocyte injury was a complex outcome for defending cell death under different pathological conditions. These postmitotic cells has limited capacity for regeneration. PECs and CoRL were candidates for the podocyte progenitor after podocyte injury.

## Introduction of PECs

### PECs in glomerular development

PECs emerge late during nephrogenesis. Presumptive PECs and podocytes share a common phenotype until the S-shaped body stage after the mesenchymal to epithelial differentiation. Between the late S-shaped body and capillary loop stage, some PECs constitute Bowman’s capsule. The others upregulate podocyte-specific genes, gradually differentiating into podocytes in mouse [5]. Once PECs differentiate into podocytes, they lose their ability to proliferate and amplify [5].

### The function of PECs

PECs serve important roles in the process of replacing injured podocytes. PECs and podocytes derived from the common mesenchymal progenitors and ultimately develop different phenotypes during glomerulogenesis [12]. Studies have identified CD133^+^CD24^+^ cells located in Bowman’s capsule that were capable of podocyte and tubular differentiation in vitro [6, 8]. Some PECs coexpress proteins unique to both podocytes and PECs [13–16]. Lasagni et al*.* demonstrated that PEC differentiation into podocytes can be enhanced by the glycogen synthase kinases 3-α and 3-β (GSK3) inhibitor BIO (6-bromo-indirubin-3’-oxime) both in vivo and in vitro. Furthermore, BIO could increase retinoic acid (RA) binding to its specific RA response elements (RARE) and strengthen PEC sensitivity to the differentiation effect of endogenously produced RA.

PECs show hierarchical differentiation based on their location. At urinary pole, PECs express CD133 and CD24 without podocyte markers (nestin, complement receptor-1, and podocalyxin), which were defined as CD133^+^CD24^+^PDX^−^ PECs; In the same way, they were defined as CD133^−^CD24^−^PDX^+^ PECs in the vascular pole and CD133^+^CD24^+^PDX^+^ PECs in the rest of regions [8]; In the case of progressive podocyte depletion, PECs begin to express podocyte proteins, including those related to focal segmental glomerular sclerosis (FSGS), aging nephropathy, and membranous nephropathy [13, 14]. PECs expressing CD133 and CD24 can alleviate renal damage by promoting tubular regeneration and podocyte substitution. They can also induce glomerular injury, such as crescent formation and glomerulosclerosis [17, 18]. Several studies have shown that the invasion of activated PECs contribute to FSGS [19]. Adhesion of the glomerular tuft to Bowman’s capsule occurs in the early stage of FSGS development, serving as a bridge for PEC migration [20]. Hence, detecting activated PECs could be an auxiliary diagnostic method for early FSGS.

Studies have shown that PECs play an important role during normal glomerular development and normal function under healthy and disease conditions [9, 21]. First, PECs are progenitors of podocytes. Second, the intracellular tight junctions of PECs can restrict glomerular filtrate to the urinary space. Third, PECs can possibly synthesize and repair the basement membrane of Bowman’s capsule [21].

Although PECs are important for health and disease, in some disease states, the reaction of PECs directly contributes to the deterioration of glomerular function. On the one hand, the abnormal proliferation of PECs could lead to crescentic glomerulonephritis and collapsing glomerulopathy. Proliferation of PECs leads to a substantial increase in cell number within crescents in murine nephrotoxic serum nephritis and collapsing glomerulopathy [17]. On the other hand, PECs are involved in glomerular scarring [22]. It is currently believed that extracellular matrix accumulation likely arises from activated PECs, and podocyte injury is the mechanism of segmental scarring in FSGS [23, 24]. The functions of PECs are summarized in Table 1.

**Table 1:** The functions of PECs

Useful functionality	Harmful functionality
1. Progenitor function for podocytes	1. Abnormal proliferation leads to crescentic glomerulonephritis and collapsing glomerulopathy
2. Intracellular tight junctions restrict glomerular filtrate to urinary space	2. Participate in glomerular scarring
3. Possible synthesis and repair of Bowman’s basement membrane	

## PECs can transdifferentiate toward podocytes

### Under physiological conditions

In juveniles, PECs can migrate to the vascular pole and differentiate into mature podocytes. Appel et al. [25] generated triple-transgenic juvenile mice that allow irreversible and specific labeling of PECs through administration of doxycycline for 14 days. They observed that all genetically labeled cells coexpressed podocyte marker proteins. Wanner et al. [26] genetically labeled PECs with membrane-tagged enhanced green fluorescent protein (mG) in inducible *hPODXL1.rtTA;tetO-Cre;mT/mG* mice exposed to doxycycline from embryonic day (E) 8.5 to postnatal day (P) 28. Interestingly, mG-labeled podocytes were observed in mouse kidney sections after postnatal kidney development. Additionally, the PEC-derived podocytes were measurable in P1 kidneys.

Interestingly, the number of PEC-derived podocytes gradually increased with age in animals [27]. In the mature kidney, PECs can serve as precursor cells to differentiate into mature podocytes and supplements to podocyte deletion. PECs can be divided into three subtypes according to their location as mentioned above. Injection of CD133^+^CD24^+^PDX^−^ cells, but not CD133^−^CD24^−^PDX^+^ or CD133^+^CD24^+^PDX^+^ cells, into mice with Adriamycin-induced nephropathy reduced proteinuria and improved chronic glomerular damage [8]. Then, Kietzmann et al*.* established an immortalized polyclonal human PEC line [28]. These researchers observed that human PECs highly expressed PEC-specific markers but did not express or weakly expressed podocyte-specific markers. Using a preclinical model of FSGS, Schneider et al*.* showed that the podocyte density was lower in aged mice than in young mice. However, the percentage of activated PECs was higher in aged mice [27]. Additionally, the percentage of phosphorylated ERK (pERK)-stained PECs was highest in aged FSGS mice, suggesting that phosphorylated pERK might be a potential mechanism needing further exploration.

In summary, PECs can migrate to the vascular pole and differentiate into mature podocytes in juvenile kidneys. Additionally, PECs can serve as precursor cells to differentiate into podocytes and supplements to podocyte deletion in mature kidneys. However, is the replacement of podocytes by PECs sufficient in aged kidneys? Podocyte numbers were lower in aged mice than young mice. The capacity for PEC-to-podocyte transition was reduced with aging. This is a multifactorial process, and the cell fate of PECs is multivariable and in need of thorough study.

### Under pathophysiological conditions

Podocyte injury and deletion, which lead to PEC activation and matrix secretion, are key factors in glomerular sclerosis [29–31]. Many glomerular diseases, such as FSGS and diabetic nephropathy (DN), are associated with podocyte injury.

FSGS is characterized by initial injury to podocytes, with secondary activation in the neighboring glomerular PECs. Smeets et al*.* demonstrated the function of PEC-producing matrix proteins by utilizing PEC-reporter mice, which contribute to the segmental scarring process [19]. These researchers demonstrated that PECs were activated following primary podocyte injury in mice. The extracellular matrix proteins produced by activated PECs result in thickening of the Bowman’s basement membrane. Similar observations in human biopsies have also been reported [32]. Thus, the mechanisms of segmental scarring in FSGS may be activated PECs followed by podocyte injury.

Glomerular PECs can serve as the progenitor niche of podocytes, which involved in the regression of DN [9]. Activated PECs are increased in patients with DN, especially in advanced stages. Podocyte populations can regenerate, which is linked to the regression of DN; concurrent reversal of DN is an attainable goal. PECs could be a progenitor cell population for the restoration of podocytes in DN [33, 34]. In the late phase of diabetes, the endothelium is terrible injured, which might lead to leakage of plasma and subsequently induce PECs activation [9].

Interestingly, the remission of podocyte loss is closely related to the differentiation of PECs into podocytes [33, 35, 36]. When many podocytes are lost, PECs can migrate to the capillary loops, and phenotypic changes occur. PECs are activated in the early stage of disease, which promotes glomerular sclerosis. During the recovery period, they differentiate into podocytes, replenish injured podocytes and promote disease repair.

After podocyte injury, the number of podocyte can be partially or completely recovered without podocyte proliferation. There are two major candidate progenitor sources for podocyte: PECs and CoRL. Circulating and bone marrow derived progenitors seemed to be out of this role. Some studies [8, 25] strongly supported that some PECs served as podocyte progenitors, while some studies [37, 38] suggested a different paradigm. They showed that the cells co-expressing PECs and podocytes specific markers at the Bowman’s capsule is that they originate from migrating cells of podocyte, not PECs. The expression of podocyte-specific proteins in PECs could be associated with protein degradation [39]. We propose a model that PECs progenitors mainly located in the tubular pole, and CoRL primarily situated in the vascular pole. However, their functions are not limited by their anatomic situations.

Finally, one might be puzzled that whether the replacement of PECs-derived podocytes is sufficient after podocyte loss? Although a subtype of PECs might differentiate into podocytes, the magnitude of regeneration may not fully replace with the depletion of podocyte [40]. The fact that the decreased number of podocyte in disease suggest that the ability of the potential podocyte progenitors to restore podocyte loss is quite restricted. We assumed that mechanism of transition are multi-factorial. When the podocyte injury is persistent and severe, the regeneration is not observed, and the matrix production and hyperplastic cellular lesion do occur, which finally lead to glomerulosclerosis and FSGS.

## Mechanism involved in the transition of PECs to podocytes

The mechanisms involved in the process of PEC-to-podocyte transition are partially understood, and observations are continually emerging [28, 41]. A better understanding of these factors could provide novel horizons for clinical therapeutics. We sum up the major signals as follows.

### Notch signaling

The Notch receptor family is evolutionarily conserved [42, 43]. Upon ligand binding, the Notch receptor initiates a series of proteolytic cleavage events, ultimately inducing the synthesis of Notch target genes.

Notch signaling regulates cell differentiation in kidney development [44]. Blockade of Notch signaling significantly alleviated PEC hyperplasia in a mouse model of FSGS [45]. Additionally, inhibition of Notch signaling suppressed the migration and mesenchymal phenotypic transition of cultured PECs, indicating that Notch-regulated mesenchymal phenotypic transformation and cell migration could replenish podocyte deficiency [46].

The podocyte loss-induced Notch activation in PECs requires further research, and TGF-β may be a candidate [45]. Properly regulating Notch expression might provide a novel strategy for the treatment of renal disease.

### Wnt/β-catenin signaling

Wnt/β-catenin signaling is a widely recognized pathway that mediates cell proliferation and differentiation, inflammation, angiogenesis, tumorigenesis and fibrosis. The activity of Wnt/β-catenin is necessary for lineage specification during the late stages of nephrogenesis [47, 48], and the Wnt pathway is activated in injured glomeruli. Activated Wnt/β-catenin signaling leads to decreased podocyte differentiation markers and increased PEC-specific markers, whereas β-catenin deletion promotes podocyte marker expression [49, 50].

Thus, the Wnt/β-catenin signal is likely involved in the transition from PECs to podocytes. The specific mechanisms require further investigation.

### Wilms’ tumor 1 (WT1)

Emerging studies have demonstrated that WT1 mutations are associated with complex developmental syndromes involving the kidney [51–53]. As a transcription factor, WT1 is required for normal renal development [52]. In adults, WT1 expression is extremely high in renal podocytes and lower in PECs [54]. WT1 null mice are unable to form kidneys [55], and WT1 mutations lead to a number of human renal diseases [56, 57].

Upregulated WT1 serves important roles in the differentiation of PECs toward podocytes [58, 59]. In addition, WT1 inhibits Wnt/β-catenin signaling [60], which is considered a prerequisite for the differentiation of PECs to podocytes.

### MiR-193a

MicroRNAs are noncoding RNAs that are approximately 21 nucleotides long and play an important role in RNA silencing by regulating mRNA degradation and protein translation. MicroRNAs are widely involved in glomerular disease.

Gebeshuber et al. [61] suggested that upregulated miR-193a in mice decreased WT1 and markers of podocytes, leading to FSGS. Leonie et al.[28] generated a human immortalized PEC line that highly expressed PEC-specific markers. Additionally, down-regulating the expression of miR-193a in human PECs resulted in transdifferentiation toward podocyte, accompanied by increased levels of podocyte markers and decreased levels of PEC markers. Interestingly, miR-193a is widely expressed in human and mouse crescents. Inhibiting the expression of miR-193a in a rodent model of nephrotoxic nephritis could decreased proteinuria and crescent formation. In addition, a luciferase assay suggested a putative interaction between miR-193a and apolipoprotein L1 (APOL1) [62]. APOL1 expression and downregulation of miR-193a coincided with the expression of podocyte markers during the transition [62], and APOL1 and miR-193a share a reciprocal feedback relationship.

Thus, miR-193a represents a master switch that modifies the expression of PEC and podocyte markers in vitro and might also be relevant in vivo.

### Growth arrest-specific protein 1 (Gas1)

Gas1 is a pleiotropic protein with multi-functions, including antiproliferative and proapoptotic activities. In kidney, Gas is expressed during nephrogenesis, and its expression is regulated by WT1 [63, 64]. Diabetes favors a decrease in Gas1 expression and increased progenitor cell markers as well as WT1 in Bowman’s capsule cells [65]. In addition, Gas1 deficiency in renal injury in the early stages of diabetes promotes the activation and proliferation of PECs, and they differentiate into podocytes [65]. Hence, Gas1 might be a novel regulator of renal regeneration in diabetes.

Above all, the Notch signaling, Wnt/β-catenin signaling, WT1, miR-193 and Gas1 were involved in the transition of PECs to podocytes. The relationship among these regulators are often neglected, which should be pay great attention: (1) β-catenin and WT1. β-catenin and WT1 are main master regulators which exhibits opposite functions in podocyte injury. Kim and colleague identified a gene, CXXC5, as a novel WT1 transcriptional target [66]. WT1 negatively controls Wnt/β-catenin pathway via CXXC5 in nephrogenesis. As study went deeper, Zhou et al. showed that β-catenin could target WT1 for ubiquitin-regulated degradation of protein, and the decreased of WT1 further in turn activated β-catenin [67]. The balance between β-catenin and WT1 could determine the state of podocyte. (2) Notch and WT1. Asfahani et al. suggested that loss of WT1 in mature podocytes regulated podocyte Notch activation, which control early events in WT1-associated glomerulosclerosis [68]. (3) Gas1 and WT1. Gas 1 is expressed in nephrogenesis and could be regulated by the transcription factor WT1.

The regeneration of terminally differentiated podocytes is a novel frontier. Accumulating evidence suggested that mechanisms involved in the process of PECs to podocyte transition are complex. The interplay among these stimulating factors for the recruitment of podocytes from PECs still need further research. An improved understanding of the mechanism is beneficial for the development of targeted drug in clinic.

## PECs as a potential therapeutic target

Numerous studies have shown that targeting PECs might have some therapeutic value [7, 69, 70]. Administering retinoids to animal models of membranous nephropathy or FSGS increased the amount of PECs, which express podocyte proteins [16]. Thus, retinoids can enhance the progenitor capacity of PECs under certain conditions. Vitamin D, which is expressed in PECs, participating in the differentiation of PECs to podocytes in vitro [18, 71]. Additionally, Notch inhibitors can modulate glomerular regeneration in animal models of FSGS, which influences glomerulosclerosis and proteinuria [72].

Interestingly, the Angiotensin-converting enzyme inhibitors (ACEi) also had potential therapeutic effects. ACEi, a well-known drug for hypertension and proteinuria, could reduce the proliferation of PECs in animal models of human immune deficiency-associated nephropathy (Tg26 mice). Additionally, ACEi enhanced the ability of PECs to turn into progenitor cells in Munich Wistar Frömter rats (which develop progressive glomerular injury) [37, 73].

Prednisone limits podocyte loss by increasing regeneration by augmenting the number of podocyte progenitors in experimental FSGS mouse model [15]. And the effects were accompanied by increasing p-ERK expression.

Other potential therapeutic goals cannot be ignored, which include CXCL12 [74], epidermal growth factor (EGF) and its receptor (EGFR) [75, 76], and amino acid transporter 2 (LAT2) [77].

CXCL12 [74] is highly expressed in normal glomerular podocytes. Suppression of CXCL12 could activate PECs that integrate into glomeruli, express podocyte specific markers, and interdigitate with existing cells.

EGFR is ubiquitously expressed in PECs and podocytes. In addition, EGFR was found to be specifically expressed in human glomerulonephritis, with proliferation and dedifferentiation of these cells. In a mouse model of RPGN, EGFR deficiency in podocytes significantly alleviated RPGN and prevented renal failure and death [75].

LAT2 is upregulated in PECs and podocytes in advance of the crescent formation as well as in the crescent lesion. Seven days after LAT inhibitor administration, the crescent formation of CGN was remarkably alleviated.

## Conclusion

**Fig. 2:**
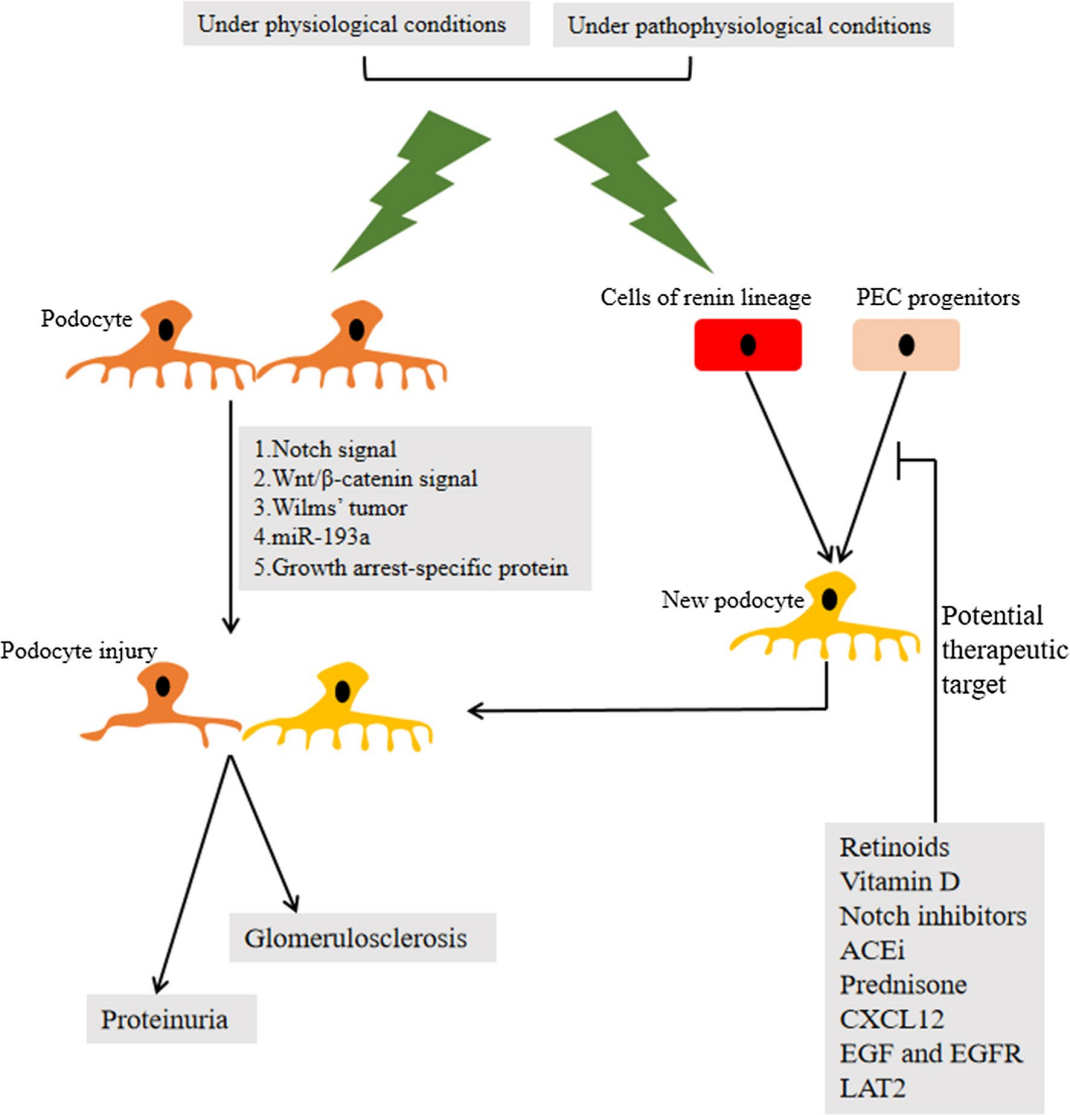
Diagram outlining the fate of podocyte under physiological and pathophysiological conditions. Meanwhile, PECs and cells of renin lineage could differentiation into new podocytes. In addition, PECs could serve as a potential therapeutic goal in podocyte injury. PECs: parietal epithelial cells

Increasing attention has been given to the biological role of PECs in health and disease as a result of PEC lineage tracing technology in animals, the exploration of specific cell markers, the isolation and identification of cultured PECs. Based on these advances, a better understanding of the potential role of activated PECs is urgent (Fig. 2).

First, replacing lost podocytes is a treating target to curb and reverse proteinuria and glomerular scarring. A subpopulation of PECs could compensate podocyte loss. However, how to identify and apply these specific PECs has been a challenge.

Second, PEC activation is manifested in both normal and disease conditions. Understanding what triggers and regulates PEC-to-podocyte transition is important and might promote repair and reduce the progression of glomerular disease. More studies are needed to improve the theoretical basis. Besides, the number of activated PECs were inadequate to fully replace the podocyte loss. The actually cues and mechanisms were worthy to be studied further.

Third, the PEC-to-podocyte transition lays the foundation for pharmacologic strategies, which aimed at accelerating podocyte, and thereby, glomerular regeneration. Novel therapeutics aiming to reduce podocyte loss or enhance PEC-to-podocyte transition might prevent glomerular disease. Further studies were needed to find new therapeutic targets for these glomerular diseases.

Fourth, PECs and CoRL are two candidates for podocyte progenitor after podocyte loss. PECs progenitors located mainly at the tubular pole of the glomerulus, while the CoRL mainly at the vascular pole. We should explore the differences between them upon podocyte injury.

Finally, there were compelling opinions that support the role of PECs as adult podocyte progenitors. We need to be cautious that there might be two-way processes that are occurred, where PECs differentiate into podocytes, and vice versa.

## Data Availability

Not applicable.
